# Analysis of *Dictyostelium discoideum* Inositol Pyrophosphate Metabolism by Gel Electrophoresis

**DOI:** 10.1371/journal.pone.0085533

**Published:** 2014-01-09

**Authors:** Francesca Pisani, Thomas Livermore, Giuseppina Rose, Jonathan Robert Chubb, Marco Gaspari, Adolfo Saiardi

**Affiliations:** 1 Medical Research Council Laboratory for Molecular Cell Biology, University College London, London, United Kingdom; 2 Department of Biology, Ecology and Earth Science, University of Calabria, Rende, Italy; 3 Laboratory of Proteomics and Mass Spectrometry, Department of Experimental and Clinical Medicine, “Magna Græcia” University of Catanzaro, Catanzaro, Italy; Université de Genève, Switzerland

## Abstract

The social amoeba *Dictyostelium discoideum* was instrumental in the discovery and early characterization of inositol pyrophosphates, a class of molecules possessing highly-energetic pyrophosphate bonds. Inositol pyrophosphates regulate diverse biological processes and are attracting attention due to their ability to control energy metabolism and insulin signalling. However, inositol pyrophosphate research has been hampered by the lack of simple experimental procedures to study them. The recent development of polyacrylamide gel electrophoresis (PAGE) and simple staining to resolve and detect inositol pyrophosphate species has opened new investigative possibilities. This technology is now commonly applied to study *in vitro* enzymatic reactions. Here we employ PAGE technology to characterize the *D. discoideum* inositol pyrophosphate metabolism. Surprisingly, only three major bands are detectable after resolving acidic extract on PAGE. We have demonstrated that these three bands correspond to inositol hexakisphosphate (IP_6_ or Phytic acid) and its derivative inositol pyrophosphates, IP_7_ and IP_8_. Biochemical analyses and genetic evidence were used to establish the genuine inositol phosphate nature of these bands. We also identified IP_9_ in *D. discoideum* cells, a molecule so far detected only from *in vitro* biochemical reactions. Furthermore, we discovered that this amoeba possesses three different inositol pentakisphosphates (IP_5_) isomers, which are largely metabolised to inositol pyrophosphates. Comparison of PAGE with traditional Sax-HPLC revealed an underestimation of the cellular abundance of inositol pyrophosphates by traditional methods. In fact our study revealed much higher levels of inositol pyrophosphates in *D. discoideum* in the vegetative state than previously detected. A three-fold increase in IP_8_ was observed during development of *D. discoideum* a value lower that previously reported. Analysis of inositol pyrophosphate metabolism using ip6k null amoeba revealed the absence of developmentally-induced synthesis of inositol pyrophosphates, suggesting that the alternative class of enzyme responsible for pyrophosphate synthesis, PP-IP_5_K, doesn’t’ play a major role in the IP_8_ developmental increase.

## Introduction

The model organism *Dictyostelium discoideum,* originally developed to study the transition to multicellularity, has subsequently been utilised in several areas of biology from chemotaxis [1] to transcriptional control [2]. Upon exhaustion of nutrients, the Dictyosteliidae slime moulds are able to aggregate into multicellular forms, a process regulated by cAMP signaling [3]. The aggregated slugs develop into fruiting bodies (or sporocarp) compromised of two main cell types; stalk cells and thousands of spore cells. Much of the early work with this amoeba focused on this fascinating behaviour. However, in the late 1980s this model organism began to offer insight into the metabolism of inositol phosphates [4]. In fact, it was in *D. discoideum* that the synthesis of inositol hexakisphosphates (IP_6_) through direct phosphorylation of inositol was discovered [5].

*D. discoideum* has also been instrumental in the discovery of inositol pyrophosphates (also known as diphosphoinositol phosphates) (For reviews see [6], [7]) molecules containing highly energetic pyrophosphate moiety(ies) recently implicated into the regulation of cellular homeostasis [8], [9], [10]. Inositol pyrophosphates were identified in 1993 in *D. discoideum* [11] and in mammalian cell [12]. During the 1990s the synthesis of the inositol pyrophosphate IP_7_ (diphosphoinositol pentakisphosphate or PP-IP_5_) and, in particular, IP_8_ (bisdiphosphoinositoltetrakisphosphate or (PP)_2_-IP_4_) was linked to the *D. discoideum* developmental program [13]. Furthermore, thanks to the high concentration of these molecules in this amoeba, NMR could be used to resolve the isomeric nature of IP_7_ and IP_8_ extracted from *D. discoideum* cells. The structure of these isoforms - the 5PP-IP_5_ isomer of IP_7_ and the 5,6(PP)_2_-IP_4_ isomer of IP_8_ [14] are, to date, the only resolved structures of inositol pyrophosphates extracted from cells.

Despite the influence of this organism, *D. discoideum* has faded from the attentions of inositol phosphate scientists over time. The last study demonstrating the importance of inositol pyrophosphate in regulating Dictyostelium chemotaxy was published over 10 years ago [15]. This disengagement is in part due to the emergence of another experimental model, the yeast *Saccharomyces cerevisiae* [16], [17] but also to the difficulty in promptly labelling the amoeba with tritium inositol (^3^H-inositol) [18]. Therefore the use of routine Sax-HPLC (Strong anion exchange chromatography) to resolve the different radiolabeled inositol phosphates becomes cumbersome and expensive to apply to amoeba cells. Thus, chromatographic separation of inositol phosphates in Dictyostelium is normally performed using metal dye detector post-colum derivatization (MDD-HPLC) [11], [19] requiring a dedicated three pump HPLC apparatus and therefore is not a widespread technology.

Two different classes of enzymes are able to synthesize inositol pyrophosphates: the inositol hexakisphosphate kinases IP6Ks (Kcs1 in yeast) [20] and the PP-IP_5_Kinases (Vip1 in yeast) [21], [22]. These enzyme are mainly characterised from mammalian sources and possess the ability to pyrophosphorylate position 5 of the inositol ring (IP6K) [23] and position 1 (PP-IP_5_K) [24] *in vitro*. Thus, it is believed that mammalian cells posses a different isomer of IP_8_, namely the 1,5(PP)_2_-IP_4_ species [25].

The recent discovery that higher inositol phosphates can be resolved by polyacrylamide gel electrophoresis (PAGE) [26] and visualised by simple staining, bypassing the need to use radio labelled material, has enormously improved *in vitro* studies of inositol pyrophosphates [27]. In particular, this has facilitated characterisation of the inositol pyrophosphate synthesizing kinases; the inositol hexakisphosphate kinases (IP6Ks in mammals, Kcs1 in yeast) and the Diphosphoinositol pentakisphosphate kinases (PP-IP5Ks in mammals, Vip1 in yeast) [26].

In the current work we applied this PAGE technology to samples obtained from live cells, allowing us to analyse the *in vivo* inositol phosphate metabolism by PAGE for the first time. We demonstrated the existence of different inositol pyrophosphate species by both DAPI and Toluidine Blue staining, and reveal a complex metabolism comprising inositol pyrophosphates derived from both IP_6_ and inositol pentakisphosphates (IP_5_). Furthermore, the analysis of inositol pyrophosphate metabolism during *D. discoideum* development revealed a far less dramatic increase in levels of IP_8_ than has been previously described.

## Results

### Resolving *D. discoideum* Cell Extract by PAGE Revealed the Presence of IP_6_, IP_7_ and IP_8_

**Figure 1:**
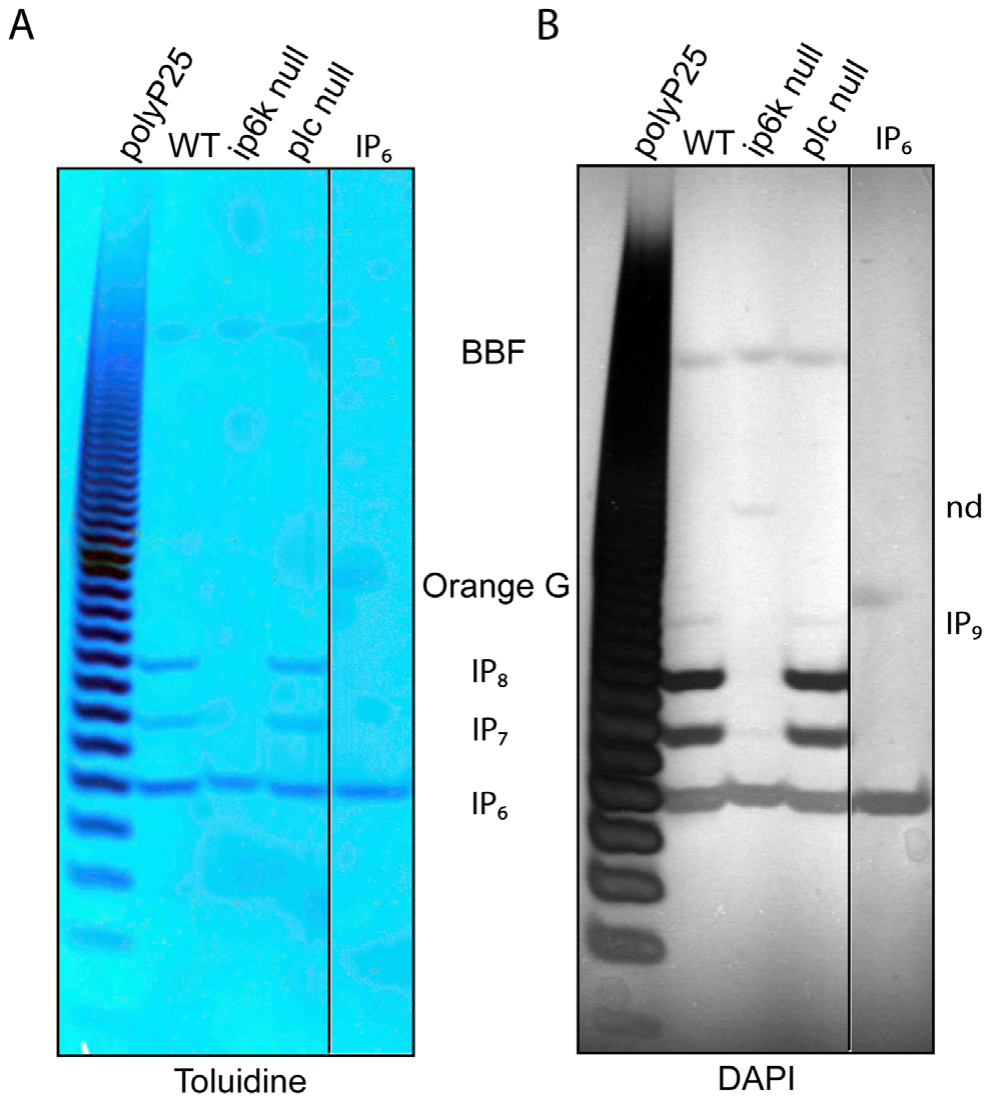
PAGE analysis of *D. discoideum* cell extract reveal the presence of three major bands. Inositol phosphates were extracted from 10(WT) *D. discoideum* (AX2 strain) and the IP_6_-Kinase (ip6k null) and phospholipase C mutant (plc null) grown at a density of 2–4×10^6^_._ About 30–40 microliters of neutralised cell extract (equivalent to 1/20 of the total volume) was resolved on 35.5% PAGE [26] and visualized with Toluidine blue (A) and DAPI staining (B). The figure shows the result of a representative experiment that was repeated three times.

Inositol polyphosphates are routinely extracted using strong acid solutions, usually Percloric Acid. If appropriately labelled with ^3^H-inositol this cell extract can be neutralised and analysed by Strong anion exchange chromatography (Sax-HPLC) [28]. The high levels of inositol pyrophosphates in *D. discoideum* prompted us to analyse a fraction (1/20 by volume) of the neutralised unlabeled cells extract, equivalent to 1–2 10^6^ cells, by PAGE [26]. Sample migration during PAGE is normal when just 20–40 microlitres of cell extract is loaded (Fig. 1). To our surprise, extract from vegetative Wild Type AX2 (WT) *D. discoideum* cells reveals the presence of three major bands by Toluidine staining. The fastest migrating band co-migrates with the commercially available IP_6_ standard (Fig. 1). Staining with DAPI also reveals the same three major bands (Fig. 1). Interestingly, DAPI is heavily photobleached (resulting in negative staining) by the two slower migrating bands and not by that which comigrates with IP_6_. This method of staining reveals a further weaker band, which migrates still slower and is not always detectable by Toluidine. It was previously demonstrated that the ability to photobleach DAPI is a typical characteristic of the pyrophosphate moiety [26], however the large amount of IP_6_ present in *D. discoideum* extract is able to induce some DAPI photo-bleaching, though at a much lower efficiency. Thus, besides IP_6_ the other bands are expected to be IP_7_, IP_8_ and, newly detected, endogenous IP_9,_ previously identified only *in vitro* [23].

To confirm the nature of these bands we use several approaches. First genetic; the analysis of acidic extract of Inositol Hexakisphosphate Kinase (IP6K) null amoeba (gene I6KA, DDB_G0278739) [15] reveals only the presence of a band co-migrating with IP_6_ and a weaker band, detectable only by DAPI, co-migrating with IP_7_ (Fig. 1). The virtual absence of IP_7_, and the total deficiency of the respective IP_8_ and IP_9_ bands phenocopies the yeast ip6k mutant (*kcs1*Δ) that lack any detectable inositol pyrophosphates [16], [29]. Using traditional ^3^H-inositol labelling and Sax-HPLC analysis, the absence of any inositol pyrophosphates in ip6k null amoeba has been previously verified [15]. Interestingly, DAPI analysis reveals the presence of a new, retarded band in ip6k null amoeba (labelled nd in Fig. 1B). This band is of undetermined nature although DAPI photobleaching ability suggests the presence of pyrophosphate moieties. The analysis of phospholypase C (PLC) mutant cells reveals a pattern of bands similar to WT cells in striking contrast of the yeast *plc1*Δ strain that lacks the synthesis of any highly phosphorylated forms of inositol phosphates [17]. However, our result is coherent with previous reports that demonstrate normal levels of inositol pyrophosphates in *D. discoideum* plc null cells (gene pipA, DDB_G0292736) [18], [30] and with the ability of the amoeba to synthesize IP_6_ directly from inositol independently from lipid cleavage [5].

**Figure 2:**
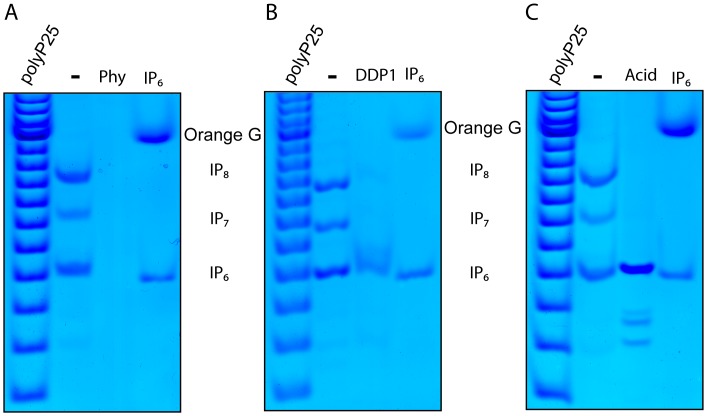
Treatment by *Phytase*, Ddp1 and acidic degradation define IP_6_, IP_7_, and IP_8_, in *D. discoideum* cell extract. Wild type *D. discoideum* cell extract (-) was incubated with phytase (Phy) (A), recombinant diphosphoinositol polyphosphate phosphohydrolase (DDP1) (B) or treated with acid at high temperature (C). The inositol phosphate nature of the three major bands detectable by Toluidine stain is demonstrated by the Phytase treatment (A), an enzyme able to remove the phosphate group from any position of the inositol rings. The pyrophosphate nature of the two slower migrating bands is demonstrated by their disappearance after DDP1 treatment (B) and by the well known acidic sensitivity of the phosphoanhydride bond (C). The figure shows the result of a representative experiments repeated three to four times.

Secondly, we use enzymology to confirm the nature of these bands as genuine inositol phosphates. The treatment of WT extract with phytase, an enzyme capable of fully dephosphorylating IP_6_ (also call Phytic Acid), resulted in the complete disappearance of the three major bands (Fig. 2A). The treatment of WT extract with DDP1 (Fig. 2B), a phosphatases that specifically degrades the pyrophosphate moiety, resulted in a almost complete degradation of IP_7_ and IP_8_ with the corresponding formation of IP_6_.

**Figure 3:**
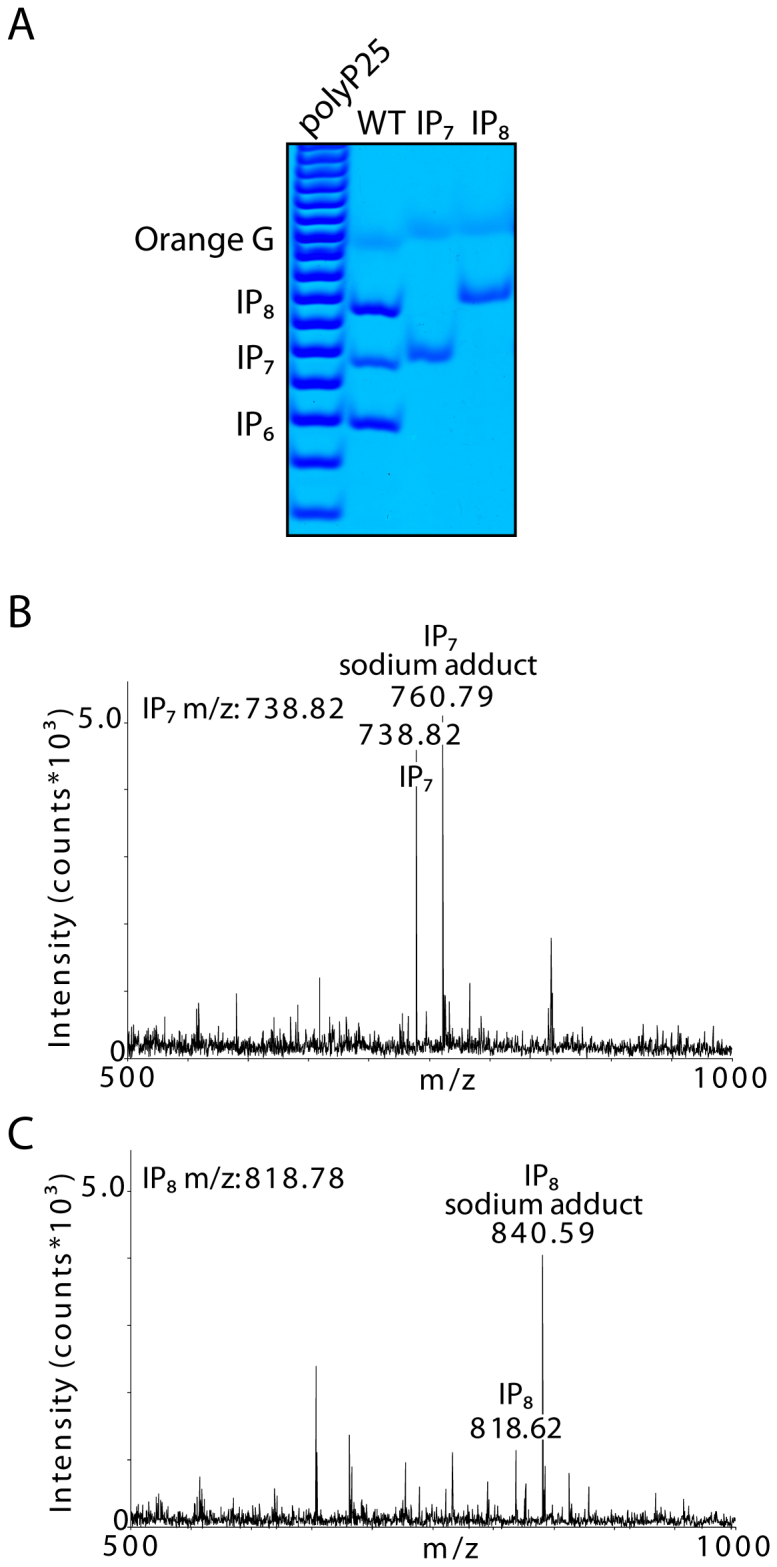
Mass spectrometry analysis of inositol pyrophosphates purified from *D. Discoideum* cell extract. Gel purified inositol pyrophosphates (A) were subjected to mass spectrometry (B,C). The comparison of the m/z spectrum of IP_7_ (B) and IP_8_ (C) is shown. The peaks in the spectra describing inositol pyrophosphates purified from *D. discoideum* are in agreement with the theoretical values for molecular weight that are deduced to be 738.82 Da and 818.78 Da respectively.

Phosphoanhydride bonds (the pyrophosphate moiety) are rapidly hydrolysed in acid at higher temperatures. Consequently, we also incubated the acidic extract at 90°C for 10 minutes before neutralization. This treatment (Fig. 2C) revealed the complete degradation of the IP_7_ and IP_8_ bands and the resultant formation of IP_6_ as well as three further fast migrating bands. These three extra bands migrate as expected of IP_5_, which is almost undetectable in untreated cell extract (Fig. 1, 2, 3). This suggests the existence of an elaborate inositol pyrophosphate metabolism (see below).

Finally we use mass spectrometry to determine the mass of the purified bands (Fig. 3A). The analysis of the putative IP_7_ band reveals, in the *m/z* range 500–1000, two major peaks at 738.822 and 760.793 *m/z*. These *m/z* values are well in accordance with the theoretical mass of deprotonated IP_7_ and its sodium adduct respectively (Fig. 3B). The detection of an intense peak at +22 *m/z* with respect to the deprotonated molecule is an additional confirmation of the presence of phosphate moieties on the analyte. Similarly, the analysis of the putative IP_8_ band reveals a major peak at 840.590 *m/z*, and a minor peak at 818.627 *m/z* (Fig. 3C). These values correspond to the theoretical mass of, respectively, the sodium adduct of deprotonated IP_8_ and deprotonated IP_8_. The increased relative intensity of the sodium adduct is an additional confirmation of the increased number of phosphate moieties attached to the analyte in the putative IP_8_ band compared to the putative IP_7_ band.

Unfortunately, we observed a decrease in mass spectrometry ionization efficiency with increasing number of phosphate groups, as can be appreciated by comparing absolute intensities of IP_7_ and IP_8_ MALDI-TOF spectra in (Fig. 3B and C). Therefore, we were unable to determine the mass of the IP_9_ band. Nevertheless, taken together these genetic and biochemical studies prove that the bands observable in *D. discoideum* extract are bona fide inositol pyrophosphates.

### Presence of a Complex IP_5_ Derived Inositol Pyrophosphates Metabolism

**Figure 4:**
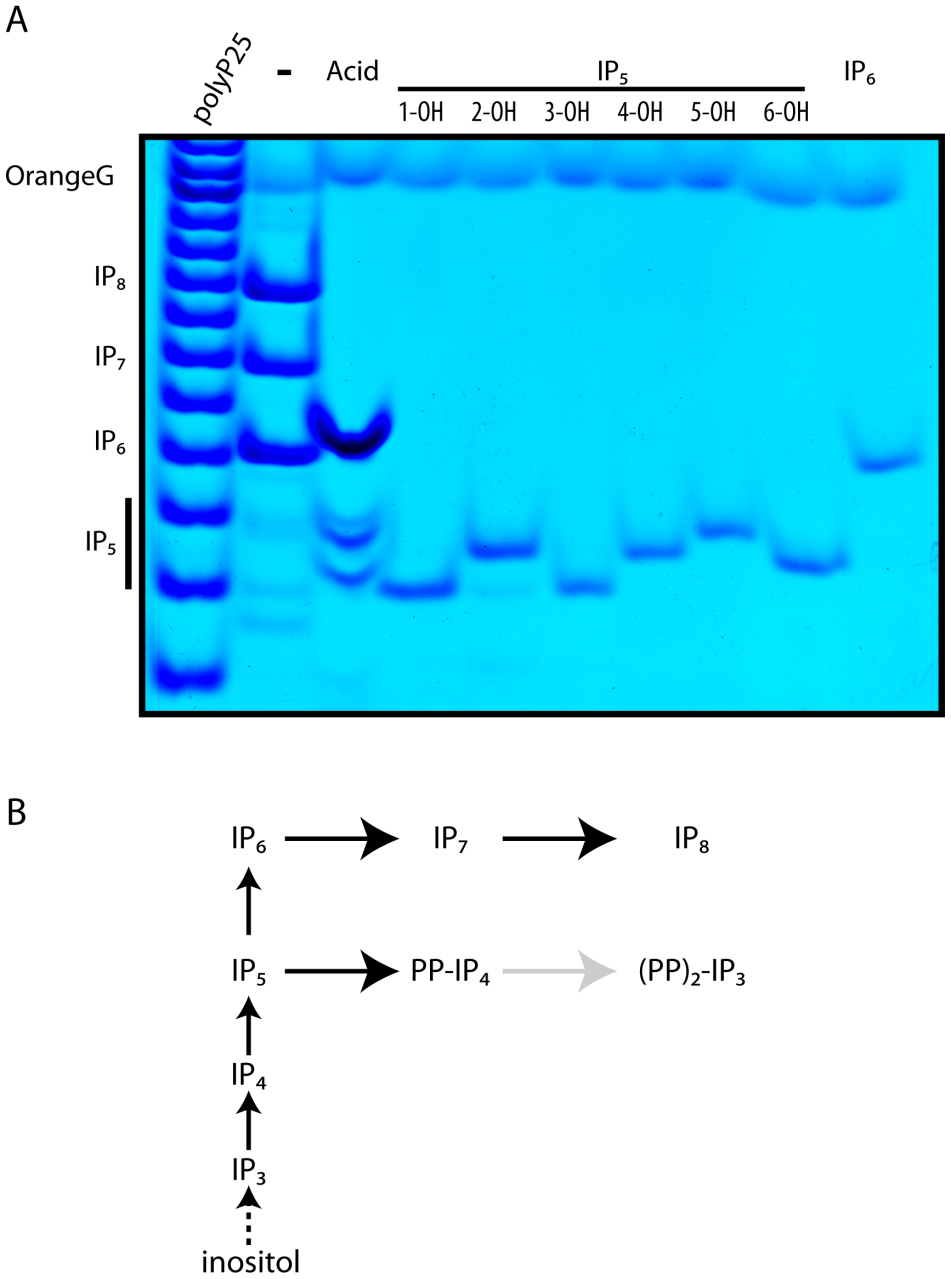
Characterization of *D. discoideum* IP_5_ species. Half of the acidic cell extract (from 5 ml culture) of WT *D. discoideum* was incubated on ice (-) while the second half was incubated at 90°C for 20 min (Acid). Both samples were thenneutralised and resolved on 35.5% PAGE together with the six possible IP_5_ isomers. Inositol phosphates were visualised by Toluidine staining. Densitometry analysis of treated versus untreated sample was performed and IP_6_ and IP_5_s bands intensity compared. (A). Acidic treatment reveals the distinct presence of three IP_5_ species, which are otherwise barely detectable, indicating that *D. discoideum* possesses a complex IP_5_-derived inositol pyrophosphate metabolism. (B) Schematic representation of inositol pyrophosphate metabolism in *D. discoideum*. The gray arrow to (PP)_2_-IP_3_ indicates a likely potentiallye step. The dashed arrow from inositol to IP_3_ indicates uncharacterized enzymatic steps. The figure shows the result of a representative experiment that was repeated three times.

The appearance of bands migrating faster that IP_6_ after acidic hydrolysis prompted us to perform further analysis with the aim to determine their exact nature. We split a cell extract (from 5 ml culture growth at a density of 2–4×10^6^ cells/ml) into two halves and subjected one half to acidic hydrolysis. After neutralisation this half as well as the untreated half were analysed by PAGE. We employed IP_6_ as a standard as well the six IP_5_ isomers (Fig. 4A). Loading a larger amount of cell extract allowed us to detect fast migrating three weak bands in the untreated sample lane. These bands co-migrate with distinct IP_5_ standards. The acidic treated samples reveals a robust increase of both IP_6_, due to the conversion of IP_7_ and IP_8_ to IP_6_, and the three IP_5_ isomers. This indicates presence of inositol pyrophosphate generated from IP_5,_ such as PP-IP_4_ and likely also (PP)_2_-IP_3_, which are converted back to IP_5_ by acidic treatment. The inositol pyrophosphates PP-IP_4_ and (PP)_2_-IP_3_ possessing six and seven phosphates groups migrate very closely (or co-migrate) with the more abundant IP_6_ and IP_7_ species and thus cannot be directly detected in untreated WT cell extract.

Densitometry analysis of treated IP_6_ and IP_5_s versus untreated counterpart reveals that ∼60% of the IP_6_ pool is converted to IP_7_ and IP_8_ while ≥90% of IP_5_ pool is converted to inositol pyrophosphate species (Fig. 4B). This indicates that pyrophosphates derived from IP_5_ and IP_6_ have differing metabolism and turnover. The extraordinary ability of PAGE to resolve different IP_5_ isomer and the densitometry analysis of the three IP_5_ bands reveal that the IP_5_ pool of *D. discoideum* cell is distributed as follows, ∼10% I(1,2,3,4,6)P_5_; ∼30% I(2,3,4,5,6,)P_5_ and/or I(1,2,4,5,6)P_5_; ∼60% I(1,3,4,5,6)P_5_ and/or I(1,2,3,5,6)P_5_ and/or I(1,2,3,4,5)P_5_. The presence of at least three IP_5_ isomers is confirmed by an early report [5], [31]. However these earlier studies, which relied on HPLC revealed differing relative distributions of the IP_5_ isomeric species [31]. It is important to note that strong acidic conditions (such as those used in HPLC analysis) can induce phosphate groups to move to adjacent hydroxyl positions, altering the isomeric nature of inositol phosphates, a well known phenomena during phosphoinositide (inositol lipid) purification [32]. High temperature and acidity are also able to induce movement of phosphate groups around the hydroxyl groups of the inositol ring in IP_5_ (Figure S1). However, the presence of the three IP_5_ species in untreated samples (Fig. 4A) is supportive of the genuine existence of at least three different IP_5_ isomers in *D. discoideum.* The fact that all of these IP_5_ species are enriched after pyrophosphate hydrolysis indicates that multiple IP_5_ isomers are precursors of inositol pyrophosphate species indicating a complex isomeric mixture of pyrophosphates derived from IP_5_.

### PAGE Analysis Revealed High Levels of Inositol Pyrophosphates

Previous studies aiming to analyse the level of inositol pyrophosphate during *D. discoideum* development have estimated the levels of IP_7_ and IP_8_ in vegetative stage cells to be 3–6% of the levels of IP_6_, a ratio comparable to that observable in mammalian cells. This data, obtained using MDD-HPLC [13], was subsequently confirmed by traditional metabolic 3H-inositol labelling and Sax-HPLC technology [15]. Strikingly, however, our extraction and PAGE analysis reveals substantially higher levels of inositol pyrophosphates during vegetative state growth (Fig. 1, 2, 3, 4). DAPI analysis reveals a markedly darker stain of the IP_8_ band over IP_6_ (Fig. 1B). This can be attributed to the favourable ability of the pyrophosphates moiety to photobleach DAPI [26]. The monoaminic Toluidine, however, stains the single phosphates groups with similar efficiency. Therefore a molecule of IP_8_ possessing 8 phosphate groups, compared to just 6 on a molecule of IP_6_ groups should stain more intensely than IP_6_. Experimentally this value has been calculated to be 1.27+/−0.08 (see material and methods for details and Figure S2). However, even taking into account this correction factor, densitometry measurement of PAGE analysis revealed the ratio of IP_8_ to IP_6_ in the vegetative state to be in the range 30–40% (Fig. 1, 2, 3, 4). Therefore, traditional HPLC technology substantially underestimates the level of cellular inositol pyrophosphates. It is likely that this effect is due to the fact that pyrophosphate (phosphoanhydride) bonds are acid labile and prone to degradation during acidic HPLC running conditions.

To further confirm this observation we ran cultures in parallel; one labelled with ^3^H-inositol and analysed by HPLC, while the second was run by PAGE and analysed by Toluidine staining. We rapidly extracted the inositol phosphates at 4**°**C to minimise the duration and effect of the acidic conditions. This parallel analysis, reveals that the IP_8_ level as ratio over IP_6_ was 27.5%+/−6.9 (n = 4) and 36.3%+/−4.7 (n = 4) analysed by Sax-HPLC or PAGE respectively. Therefore traditional HPLC analysis results in a substantial 1/4 underestimation of IP_8_ cellular levels.

Application of PAGE to this *in vivo* system for the first time allows us to determine the intracellular concentration of highly phosphorylated inositol phosphates by simple densitometry (using IP_6_ concentration standards to simply calculate a linear regression curve). Our study reveals that in vegetative state, estimating a cell volume of 0.20 pL, the concentration of IP_6_, IP_7_ and IP_8_ are ∼520, 60 and 180 µM respectively. Interestingly, the IP_6_ value is in accordance with previous estimates [5], [33].

### Inositol Pyrophosphates Cellular Levels Increase during Development

Dictyostelium development occurs upon exhaustion of food supply. This starvation response can be induced by shifting vegetative *D. discoideum* cells to agar plates made with a simple phosphate buffer (see material and methods). The regulation of inositol pyrophosphate metabolism during the slime mould’s developmental program has been previously investigated [13]. This study revealed the most dramatic cellular concentration change in IP_7_ and IP_8_ so far reported, with a 25-fold increase of IP_8_ level [13]. However, our obseravtions that IP_7_ and IP_8_ are present at high levels in vegetative cells (Fig. 1–4) led us to question the scale of this dramatic increase. Therefore we subjected WT and ip6k null cells to starvation, inducing the developmental program.

**Figure 5:**
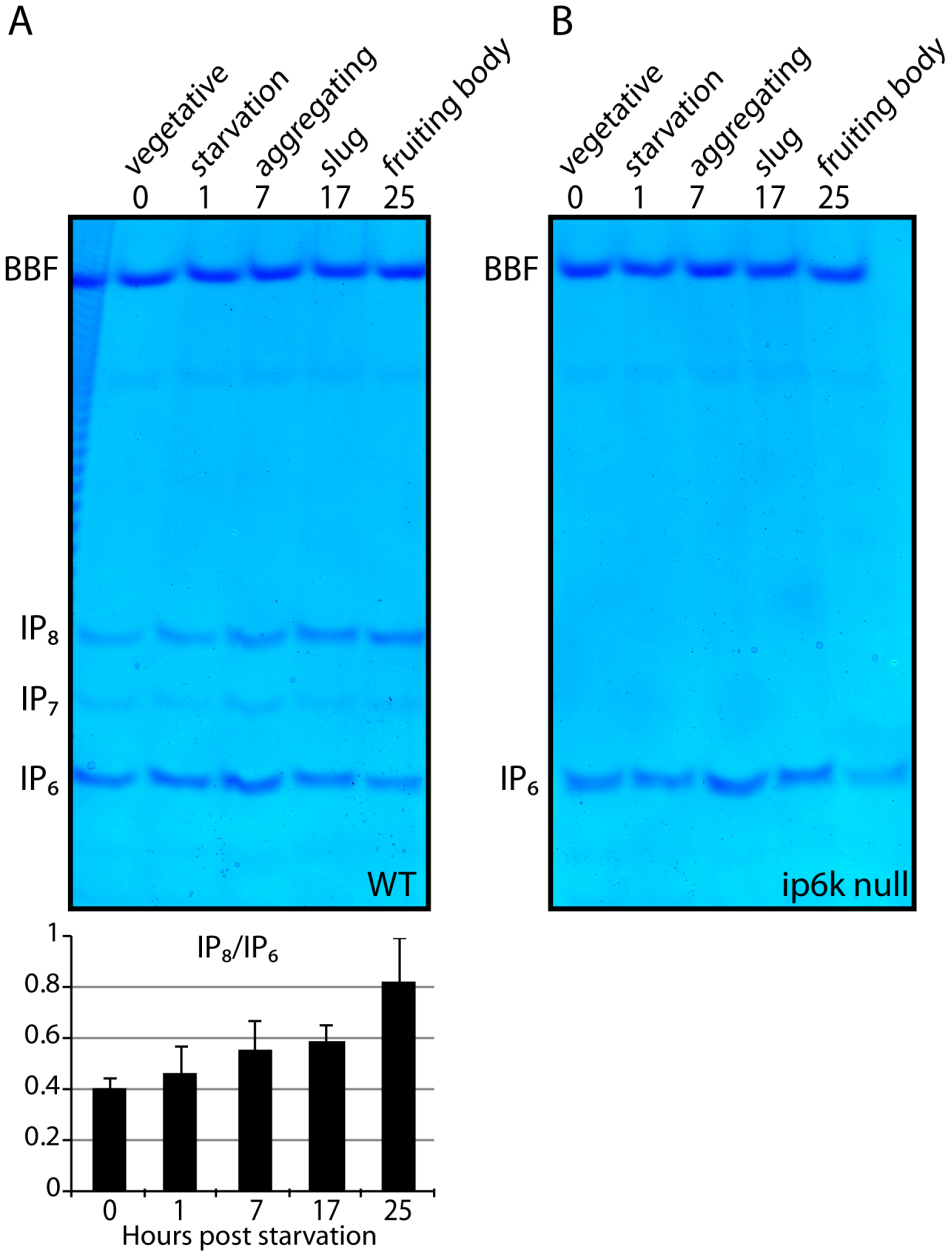
PAGE analysis of inositol pyrophosphate during *D. Discoideum* development. Amoeba development program was induced as described in material and methods. The inositol phosphates extracted at the indicated time points were resolved on 35% PAGE and visualised with Toluidine. (A) The analysis of wild type (WT) *D. discoideum* developmental program reveal a 2.6fold increase in the IP_8_/IP_6_ ratio at the late stage of development, as quantified by densitometry quantified (Bottom), average +/− SD of four independent experiments. (B) To the contrary inositol pyrophosphates are not induced during IP_6_-Kinase (ip6k null) developmental program. The figure shows the result of a representative experiment that was repeated four times for the WT and two times for ip6k1 null.

Cells were grown to a density of 2×10^6^ cells/ml, washed in phosphate buffer and then plated onto 20 mM phosphate buffer agar plates. Cells were collected at 5 time points; time zero, whilst still in the vegetative state; after one hour of starvation; upon first visual signs of aggregation (6–9 hr depending on strain); during the “slug” stage 15–17 hs after induction of starvation and finally after 24 hr as mature fruiting bodies. Analysis of acidic cell extract shows a clear increase of IP_8_ (in comparison to IP_6_) of 2,6 fold during the developmental time course. Therefore, although we observe a clear and substantial increase in IP_8_ levels (Fig. 5A), it is in the region of three fold, well below the 25-fold seen previously by HPLC analysis [13].

We also performed developmental study of ip6k null stain (Fig. 1) [15]. This analysis revealed the lack of induction of any inositol pyrophosphate forms (Fig. 5B). This data indicates that the *D. discoideum* PP-IP_5_K homologous gene (*DDB_G0284617*) does not play any major role in the developmental increase of inositol pyrophosphates.

**Figure 6:**
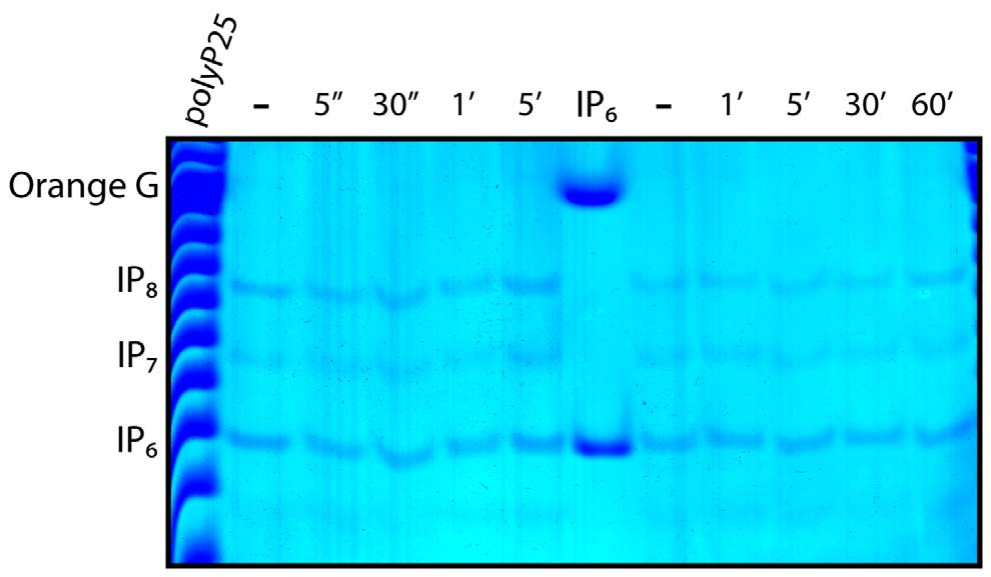
No alteration of IP_7_ and IP_8_ metabolism after cAMP treatment. Vegetative growing *D. Discoideum* were incubated for the indicated time with 50 µM cAMP. The incubation was terminated with equal volume of 2M Percloric acid to extract the inositol phosphates. These were resolved on 35% PAGE and stained with Touilidine blue. Two independent experiments are shown with short (left) and long (right) cAMP incubation time. The figure shows the result of a representative experiment that was repeated three times.

The *D. discoideum* development program is elicited by cAMP signal and it was reported that cAMP stimulation induced a rapid (within minutes) threefold increase in inositol pyrophosphate levels [15]. We repeated these studies and failed to see any significant change in IP_7_ and IP_8_ levels in response to cAMP when analysed by PAGE (Fig. 6).

## Discussion

The recently developed PAGE technology to resolve and visualise inositol phosphates has been previously employed to characterise *in vitro* enzymatic reaction [26], [27]. Here we show the huge potential of this technology to study inositol pyrophosphate metabolism in *D. discoideum*. The high abundance of this class of molecules in *D. discoideum*, coupled with the ease of analysis by PAGE has allowed us to re-evaluate the regulation of inositol pyrophosphate metabolism during the amoeba development. This re-evaluation has revealed a 3-fold increase in IP_8_ levels reached in mature fruiting bodies a value far below the 25-fold increase that was previously determined by MDD-HPLC [13]. MDD-HPLC technology requires the extracted samples to be resolved using an elution buffer containing 0.1M Hydrochloric Acid [13]. This condition is likely to result in the hydrolysis of pyrophosphate bonds and thus increased variability between samples. Therefore, the difference of IP_8_ induction during Dictyostelium development between our PAGE analysis and the previous study [13] is most likely due to the acidic sensitivity of the pyrophosphate moiety and its degradation during the strong acidic conditions associated with MDD-HPLC.

In agreement with this, analysis of ^3^H-inositol labelled inositol pyrophosphates by Sax-HPLC, a technology that requires less acidic conditions (Ammonium Phosphate buffer at pH 3.8) reveals that the IP_8_ ratio over IP_6_ is ∼27% higher than previous studies suggested [13]. However, pyrophosphate hydrolysis still occurs in the mildly acidic Sax-HPLC running conditions. In fact, when the IP_8_ ratio over IP_6_ ratio was measured by PAGE and densitometry (sample resolved in 1XTBE, buffer pH8.0) this value was still higher at ∼36%. Therefore both *in vitro* study [26] and also the current *in vivo* PAGE analysis suggest that HPLC analysis underestimates the cellular levels of inositol pyrophosphates. Unfortunately, technical problems still preclude the application of PAGE analysis to mammalian cells. As such, HPLC analysis remains, at least for now, the only viable method for this system. We have also demonstrated that PAGE can resolve several of the different IP_5_ isomeric species (Fig. 4A). This has allowed us to observe at least three different IP_5_ isomers in vegetative *D. discoideum* consistent with early reports [5], [31]. This is not a surprise as mammalian cells also possess multiple IP_5_ species [34], [35]. Unexpectedly, all the IP_5_s species are precursors of inositol pyrophosphates. Therefore, the inositol pyrophosphates derived from IP_5_ are quite complex in compositions, with multiple isomeric forms of PP-IP_4_ and (PP)_2_-IP_3_ existing *in vivo*. The same enzymes that generate IP_7_ from IP_6_, the IP6Ks, also generate these inositol pyrophosphates species *in vitro* [16], [29]. Thus the relative cellular abundance between IP_7_/IP_8_ and PP-IP_4_/(PP)_2_-IP_3_ might depend on the levels of IP_5_ versus IP_6_. While in *D. discoideum* IP_6_ is far more abundant of IP_5_ this is not the case the majority of mammalian cell lines where the cellular concentration of these two inositol polyphosphates are similar and regulated in neuron by neurotrofine signal [36]. Consequently inositol pyrophosphate derived from both IP_5_ and IP_6_ precursors are likely to have similar cellular abundance and physiological importance. More attention needs to be invested to study the functions of the IP_5_ derived inositol pyrophosphates. The fact that PP-IP_4_ is often undetected on Sax-HPLC (due to co-migration with IP_6_), is neither an indicator of its absence nor of a lack of physiological roles.

The simple, inexpensive and reliable PAGE analysis leads to clear qualitative and quantitative information by using simple densitometry. Classical ^3^H-inositol labelling and Sax-HPLC analysis of highly phosphorylated inositol perhaps retains the advantage of a higher dynamic range to calculate the relative abundance of the inositol polyphosphates. However, the use of radioactive material and HPLC apparatus has limited the implementation of inositol phosphate research to the large majority of cell biology laboratories. Here we have demonstrated the huge potential of PAGE technology to study *D. discoideum* inositol phosphate metabolism. PAGE analysis coupled with the generation of the knockout mutant strains for the several inositol phosphate kinases present in the amoeba genome will create a genetic system that will easily surpass the *S. cerevisiae* model, due to the higher complexity and greater similarity to the mammalian system.

The obvious future objective is to apply this PAGE technology to analyse inositol pyrophosphate metabolism in mammalian experimental models. However, because of the low abundance of inositol polyphosphate in mammalian cells, the direct application of PAGE technology to this system is not yet possible (Saiardi lab unpublished result). On the other hand, the effortless nature of PAGE technology should encourage further effort towards this goal, thereby opening new avenues for investigation.

## Materials and Methods

### Strains, Media and Reagents

We used the axenic *D. discoideum* strain AX2, *ip6k* null (axeA2,axeB2,axeC2,I6KA-[KO-vector],bsR) and *plc* null (axeA1, axeB1, axeC1, plc-[pNeoPLCko], neoR) background have been previously described [15], [18] and were obtained from dictyBase (http://dictybase.org). *D. discoideum* was generally grown in HL/5 or SIH acquired from Foremedium in presence of penicillin and streptomycin (Gibco). Polyacrylamides, TEMED, ammonium persulfate, were acquired from National Diagnostic. Inositol phosphates were acquired from Calbiochem (IP_6_) and Sichem (IP_5_s). All others reagents were purchased from the Sigma-Aldrich. Recombinant His-DDP1 was expressed and purified as previously described [8].

### *Culture* Condition and cAMP Treatment

Amoeba cells were inoculated at a density of 1×10^5^ cells/ml in HL5 in a glass flask, and incubated with shaking at 22°C, 120 RPM. To keep the cells in the vegetative state, stock cultures were diluted every 2–3 days such that cell density didn’t surpass 5–6×10^6^ cells/ml. Every 2–3 weeks new *D. discoideum* were started from DMSO stock. Treatment with cAMP was performed on active growing vegetative stage cell. cAMP was added to a final concentration of 50 µM to100 µl of cells and the treatments were terminate by adding 100 µl of Percloric Acid 2 M to initiate inositol phosphate extraction.

### Inositol Phosphates Extraction

The inositol polyphosphate extraction procedure is an adaptation of the yeast protocol previously described [28]. *D. discoideum* cells were collected during the exponential growth phase (1–3×10^6^ cells/ml) washed twice with KPO_4_H buffer 20 mM pH 6.0 and centrifuged at 1500 RPM on a Sorval RC-3C centrifuge for 3 min. The cell pellets were transferred to eppendorf tubes, resuspended in 1 M Perchloric acid, vortexed for 5 min at 4°C and centrifugated at 14000 RPM at 4°C for 5 min. The supernatants were transferred to a new tube and neutralised using 1M Potassium Carbonate containing 3 mM EDTA. The samples were placed on ice for 2–3 hours and subsequently spun for 10 min. The supernatants were transferred to new tubes and stored at 4°C. If required, the supernatants volume was reduced using a speed vacuum.

### PAGE Analysis and Band Intensity Analysis

To resolve inositol phosphates we used 24×16×0.1 cm glass plates, using 35% polyacrylamide in 1XTBE. Samples were mixed with 6×Dye (0.01% Orange G or Bromophenol Blue; 30% glycerol; 10 mM TrisHCl pH 7.4; 1 mM EDTA). Gels were pre-run for 30 min at 300 V and run at 600 V 6 mA overnight at 4°C until the Orange G had run through 2/3 of the gel. Gels were stained with DAPI or Toluidine Blue as described previously [26]. After scanning, the Tiff format file, band densitometry was performed using ImageJ software (http://rsbweb.nih.gov/ij/). To determine the differential Toluidine Blue staining efficiency pure IP_8_ and IP_7_ were converted to IP_6_ by acid hydrolysis and resolved by PAGE. The different densitometry intensity of IP_7_ and IP_8_ untreated samples versus the generated IP_6_ was then calculated (Supporting Fig. S2). IP_8_ is 1.27+/−0.08 (average +/− standard deviation, n = 5) times more strongly labelled that than corresponded generated IP_6_, in good accordance with the theoretical value of 1.33. To determine the amount of inositol phosphates present in *D. discoideum*, cell extracts were run together with IP_6_ concentration standards from 1 nM to 8 nM. By determining the densitometry value of the IP_6_ standard a linear regression curve was calculated. The densitometry value of the IP_6_ present in the cell extract was calculated from the linear regression curve to determine its molar amount. The values for IP_7_ and IP_8_ were calculated determining the densitometry of the respective bands normalised for the Toluidine staining efficiency (1.27 for IP_8_ and 1.15 for IP_7_). The cellular concentration of inositol phosphate was then calculated estimating a cell volume of 0.20 pL.

### Enzymatic Reactions and Acid Idrolysis

Neutralised *D. discoideum* cell extract, or purified inositol phosphate, were incubated in 30 µl enzymatic reactions containing 5XBuffer (100 mM Hepes 6.8; 250 mM NaCl; 30 mM MgSO4; 5 mM DTT; 5 mM NaF), 2 µl of recombinant purified Ddp1 (10-2-ng) or *Phytase* (Sigma). Reactions were incubated at 37°C for 2 hr or overnight and stopped by the addition of 2 µl EDTA (100 mM). Acidic pyrophosphate hydrolysis was performed by incubating the cell extract at 90°C for 20 min prior to neutralisation. After this treatment the samples were neutralised using Potassium Carbonate as described above.

### Mass Spectrometry

Inositol phosphates from *D. discoideum* cell extract were purified as described above and previously [28] and directly subjected to mass spectrometry [37]. Matrix-assisted laser desorption ionization (MALDI) mass spectrometry was performed on a Voyager DE-STR (Applied Biosystems, Framingham, MA), equipped with a MALDI ion source and a time-of-flight mass analyzer (MALDI-TOF). 9-aminoacridine (9-AA, Sigma-Aldrich) was used as matrix, due to its superior performance in revealing acidic analytes in negative ion mode [37]. A double deposition sample preparation procedure was adopted. Typically, 0.5 µL of matrix solution, consisting of 7 mg/mL 9-AA in a 1∶1 mixture (v/v) of acetonitrile and water was spotted on the stainless steel MALDI sample stage and air-dried. Then, 0.5 µL of the analyte solution, either pure or diluted 1∶5 (v/v) in water, was spotted on to the matrix crystals and allowed to dry. Mass spectra were acquired in delayed extraction, reflectron negative ion mode using the following settings: accelerating voltage 20,000 V, grid voltage 73%, extraction delay time 300 nsec, acquisition mass range 300–1,500 m/z. Each spectrum was the average of 400–500 individual laser shots acquired in series of 100 consecutive shots.

### Sax-HPLC Analysis

*D. discoideum* were cultured in inositol free SIH media containing 50 µCi/ml [^3^H]inositol. Cell culture (6 ml) were seeded at 1×10^5^ cells/ml and grown at 22°C for 3–4 days to get a cell density of 2–3×10^6^/ml. Cells were collected and washed once with KPO_4_H buffer 20 mM pH 6.0. Inositol phosphates were extracted as described above and resolved by HPLC as previously described [28].

### *D. discoideum* Development

*D. discoideum* were cultured in HL/5 media to a density of 2.0×10^6^ cells/ml The cells were washed twice with KPO_4_H buffer 20 mM pH 6.0 and resuspended at 1×10^7^ cells/ml in the same buffer Cells were then transferred in solution to 35 mm, 20 mM phosphate agar plates such that each plate contained 1×10^7^ cells. The cells were allowed to settle before aspirating the phosphate buffer. The cells were then allowed to develop in a humidity chamber at 22 C. 10 plates (equivalent to a 1×10^8^ cells at the start of the time course) were harvested from plates at 5 time points; 0 hr during vegetative state; after 1 hr starvation; upon first signs of aggregation (6–9 hr); during the “slug” stage (15 hr); and finally as mature fruiting bodies (24–25 hr). Cells pellets were frozen at −80°C. Inositol polyphosphates were extracted as described above, normalised by protein concentration and analysed by PAGE.

## Supporting Information

Figure S1 **IP_5_ isomerisation by acid treatment.** To verify that acid treatment of IP_5_ can induce movement of phosphate groups around the inositol ring we incubated two nanomols of IP_6_ and two nanomols of I(1,3,4,5,6)P_5_ with 1M Percloric acid for 30 min in ice as well as for 5 and 30 minutes at 900C. IP_6_ is totally unaffected by these treatments. Untreated I(1,3,4,5,6)P_5_ (lane 2) is 95% pure as demonstrated by its migration as a major single band. Low temperature acid treatment has no effect on I(1,3,4,5,6)P_5_, whilst high temperature induces rapid isomerisation. Just five minute at high temperature are sufficient to substantially convert I(1,3,4,5,6)P_5_ into other IP_5_ isomeric forms. Densitometry analysis confirmed that the total IP_5_ Toluidine staining did not change upon acid treatment, indicating the absence of acid-induced IP_5_ degradation to lower inositol phosphates.
(PDF)

Figure S2 **Differential Toluidine Blue staining capability of IP_8_ and IP_6_.** To ascertain the relative efficiency of staining of IP_8_ and IP_6_ by Toluidine blue serial amounts of IP_8_ from 1 nmol (A) to 16 nmol (E) were incubated in the presence of 1M Percloric acid in 20 ml (sample from A′ to E′) for 30 min at 90°C. Untreated (from A to E) and acid-treated (from A′ to E′) samples were resolved on 35% PAGE. To avoid loss of material during the neutralization step, acid-treated samples were directly loaded on the gel causing a slight retardation in migration in these lanes (as shown by the different migration of Bromophenol blue (BBF) between treated and untreated samples). Once stained with Toluidine blue, the gel was analysed with ImageJ software. Densitometry analysis enabled each pair of samples (treated and untreated) to be plotted on a graph. The areas of the peaks in these graphs correspond to the relative staining of the IP_6_ and IP_8_ bands on the gel. Depicted are the analyses of samples D–D′ and E–E′. Dividing the densitometry derived values for untreated IP_8_ by those for the acid-generated IP_6_ indicates the difference in staining efficiency of the two molecules by Toluidine blue. On average IP_8_ is stained 1.27+/−0.08 (+/− SD) better that IP_6_. A virtually identical result was obtained from a second, independent experiment also run in quintuplicate. The experimentally calculated value of 1.27 is in good accordance with the theoretical value of 1.33 reflecting the presence of eight phosphates groups in IP_8_ rather than the six in IP_6_ (8/6 = 1.33).
(PDF)
