# Supplementary material for: Analysis of Dictyostelium discoideum Inositol Pyrophosphate Metabolism by Gel Electrophoresis
Source: PLoS One. 2014 Jan 9;9(1):e85533. doi: 10.1371/journal.pone.0085533 (PMC3887064; doi:10.1371/journal.pone.0085533)
Supplement: Figure S1 — IP5 isomerisation by acid treatment. To verify that acid treatment of IP5 can induce movement of phosphate groups around the inositol ring we incubated two nanomols of IP6 and two nanomols of I(1,3,4,5,6)P5 with 1M Percloric acid for 30 min in ice as well as for 5 and 30 minutes at 900C. IP6 is totally unaffected by these treatments. Untreated I(1,3,4,5,6)P5 (lane 2) is 95% pure as demonstrated by its migration as a major single band. Low temperature acid treatment has no effect on I(1,3,4,5,6)P5, whilst high temperature induces rapid isomerisation. Just five minute at high temperature are sufficient to substantially convert I(1,3,4,5,6)P5 into other IP5 isomeric forms. Densitometry analysis confirmed that the total IP5 Toluidine staining did not change upon acid treatment, indicating the absence of acid-induced IP5 degradation to lower inositol phosphates. (PDF) [file pone.0085533.s001.pdf]

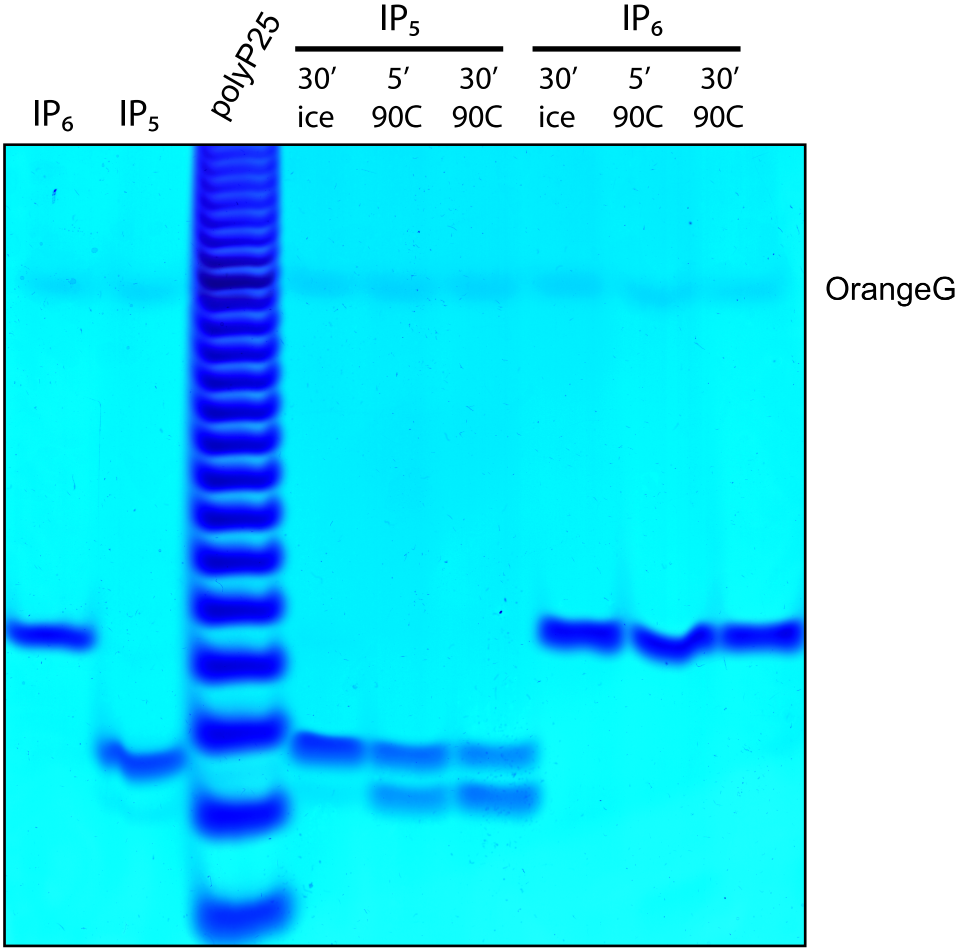

Figure S1. IP_5 isomerisation by acid treatment.
To verify that acid treatment of IP_5 can induce movement of phosphate groups around the inositol ring we incubated two nanomols of IP_6 and two nanomols of $I(1,3,4,5,6)P_5$ with1M Percloric acid for 30 min in ice as well as for 5 and 30 minutes at 90^0C. IP_6 is totally unaffected by these treatments. Untreated $I(1,3,4,5,6)P_5$ (lane 2) is 95% pure as demonstrated by its migration as a major single band. Low temperature acid treatment has no effect on $I(1,3,4,5,6)P_5$, whilst high temperature induces rapid isomerisation. Just five minute at high temperature are sufficient to substantially convert $I(1,3,4,5,6)P_5$ into other IP_5 isomeric forms. Densitometry analysis confirmed that the total IP_5 Toluidine staining did not change upon acid treatment, indicating the absence of acid-induced I P_5 degradation to lower inositol phosphates.
