# Supplementary material for: Analysis of Dictyostelium discoideum Inositol Pyrophosphate Metabolism by Gel Electrophoresis
Source: PLoS One. 2014 Jan 9;9(1):e85533. doi: 10.1371/journal.pone.0085533 (PMC3887064; doi:10.1371/journal.pone.0085533)
Supplement: Figure S2 — Differential Toluidine Blue staining capability of IP8 and IP6. To ascertain the relative efficiency of staining of IP8 and IP6 by Toluidine blue serial amounts of IP8 from 1 nmol (A) to 16 nmol (E) were incubated in the presence of 1M Percloric acid in 20 ml (sample from A′ to E′) for 30 min at 90°C. Untreated (from A to E) and acid-treated (from A′ to E′) samples were resolved on 35% PAGE. To avoid loss of material during the neutralization step, acid-treated samples were directly loaded on the gel causing a slight retardation in migration in these lanes (as shown by the different migration of Bromophenol blue (BBF) between treated and untreated samples). Once stained with Toluidine blue, the gel was analysed with ImageJ software. Densitometry analysis enabled each pair of samples (treated and untreated) to be plotted on a graph. The areas of the peaks in these graphs correspond to the relative staining of the IP6 and IP8 bands on the gel. Depicted are the analyses of samples D–D′ and E–E′. Dividing the densitometry derived values for untreated IP8 by those for the acid-generated IP6 indicates the difference in staining efficiency of the two molecules by Toluidine blue. On average IP8 is stained 1.27+/−0.08 (+/− SD) better that IP6. A virtually identical result was obtained from a second, independent experiment also run in quintuplicate. The experimentally calculated value of 1.27 is in good accordance with the theoretical value of 1.33 reflecting the presence of eight phosphates groups in IP8 rather than the six in IP6 (8/6 = 1.33). (PDF) [file pone.0085533.s002.pdf]

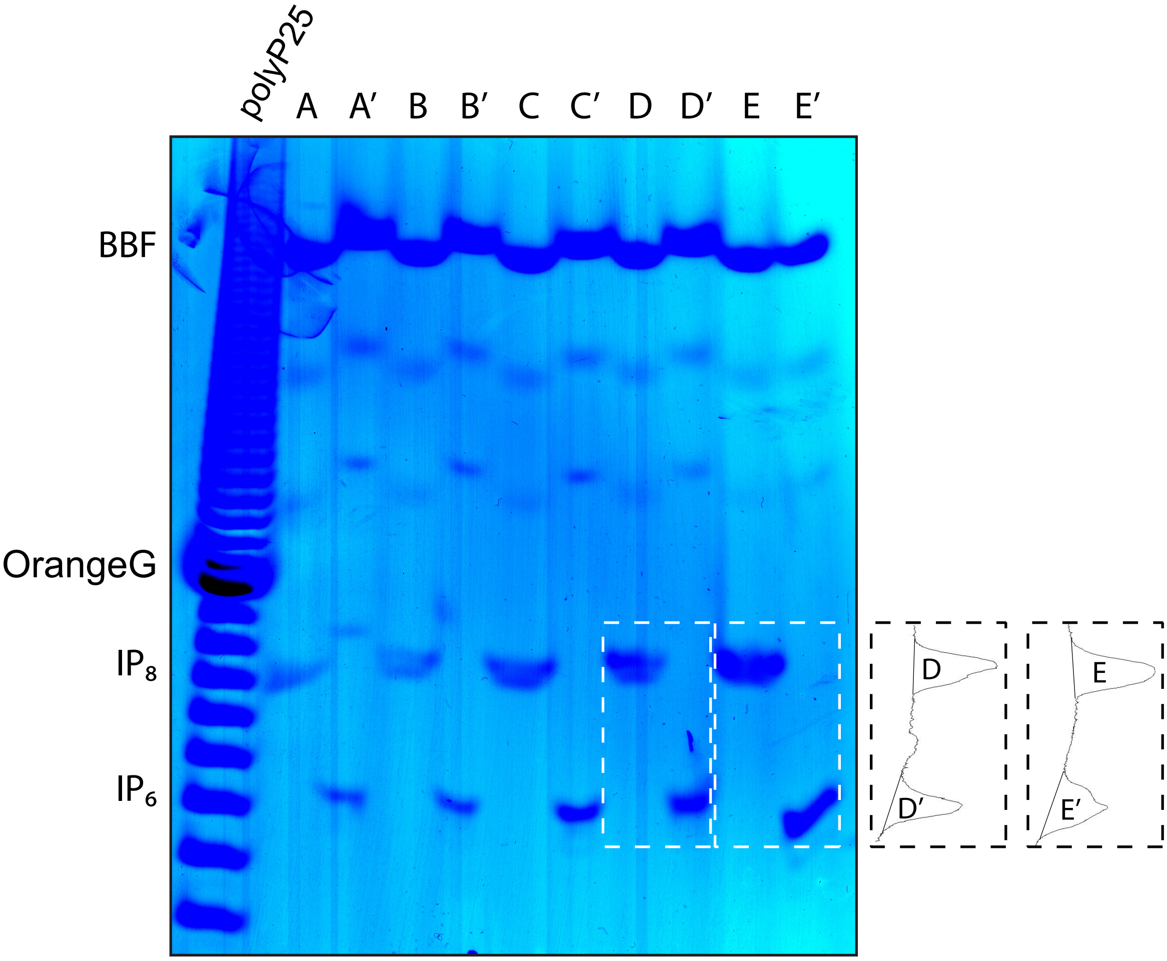

Figure S2. Differential Toluidine Blue staining capability of IP_8 and IP_6.
To ascertain the relative efficiency of staining of IP_8 and IP_6 by Toluidine blue serial amounts of IP_8 from 1nmol (A) to 16nmol (E) were incubated in the presence of 1M Percloric acid in 20μl (sample from A′ to E′) for 30 min at 90^{0}C. Untreated (from A to E) and acid-treated (from A′ to E′) samples were resolved on 35% PAGE. To avoid loss of material during the neutralization step, acid-treated samples were directly loaded on the gel causing a slight retardation in migration in these lanes (as shown by the different migration of Bromophenol blue (BBF) between treated and untreated samples). Once stained with Toluidine blue, the gel was analysed with ImageJ software. Densitometry analysis enabled each pair of samples (treated and untreated) to be plotted on a graph. The areas of the peaks in these graphs correspond to the relative staining of the IP_6 and IP_8 bands on the gel. Depicted are the analyses of samples D-D′ and E-E′. Dividing the densitometry derived values for untreated IP_8 by those for the acid-generated IP_6 indicates the difference in staining efficiency of the two molecules by Toluidine blue. On average IP_8 is stained 1.27+/-0.08 (+/- SD) better that IP_6. A virtually identical result was obtained from a second, independent experiment also run in quintuplicate. The experimentally calculated value of 1.27 is in good accordance with the theoretical value of 1.33 reflecting the presence of eight phosphates groups in IP_8 rather than the six in IP_6 (8/6 =1.33).
